# Exact Method of Locating the Apical Foramen*Read before the Michigan State Dental Society, April 8-12, 1918, and reprinted from *Journal N. D. A.*, August, 1918.

**Published:** 1918-09

**Authors:** L. E. Custer

**Affiliations:** Dayton, Ohio


### EXACT METHOD OF LOCATING THE APICAL FORAMEN *

BY L. E. CUSTER, AM., D.D.S., DAYTON, OHIO

Without doubt the most vital question before the dental profession today is, what shall we do with the pulpless tooth? The medical profession is today claiming that the teeth are the cause of all the human ills that can not be otherwise diagnosed, and some physicians have even gone so far as to advocate the extraction of all such teeth. That pulpless teeth are frequently the cause

* Read before the Michigan State Dental Society, April 8-12, 1918, and reprinted from *Journal N. D. A.*, August, 1918.

of systemic disturbances is not questioned, for that has been clearly demonstrated time and again, but we can not agree that they are so in all cases.

The seat of the whole trouble lies at the apical foramen. The problem has been to sterilize and seal this opening in a manner acceptable to nature and if the surgeon has for years been able to seal into the anatomy of a human being a piece of boiler plate the size of a man's hand or suture him up with ten feet or more of silver wire, or if the soldier returns from battle practically water logged with lead bullets, which do no harm, is it not about time that the dentist is able to seal with a foreign material so tiny an opening as the apical foramen in a manner acceptable to nature?

The whole problem involved in saving pulpless teeth and putting them in such condition that they may not become foci of infection, centers in the apical region, and as before stated, more particularly at the foramen itself. Nowhere in the whole range of human endeavor has there been as much energy spent to patch up a human being as the tiny apical foramen.

The successful treatment of pulpless teeth depends upon three procedures: first, the complete removal of all pulp tissue; second, the thorough sterilization of the dentin and periapical region, and third, the sealing of the apical foramen under aseptic conditions with a permanent non-irritating material.

The aim of this paper is very small as compared with the entire scope of pulp canal treatment, but it is just as important as any other step in the work. Pulp canals have been half filled and overfilled for want of accurate knowledge of root length. The exact location of the apical foramen is not readily determined even in the straight roots. Heretofore the sense of touch has been relied upon and in some cases the X-ray of a root with a bent wire contained therein has been the most reliable, but even this method when used is good only for the tooth rayed.

What I have here to present is two new methods of locating the apical foramen. While it is true that the foramen is not always at the extreme apex as Callahan has shown, and there may be more than one foramen to the root, the one method to be presented will meet this condition with precision and the other sufficiently so for all practical purposes. The first is an electrical method of extreme delicacy and accuracy and the other is a technic employing the X-ray with specially designed pulp canal root meters. At present I am only using these upon single rooted teeth.

The electrical method is based upon the difference in the electrical conductivity of a dry pulp canal or one filled with a non-conducting liquid, and the conductivity of the tissues just beyond the apical foramen. That is, the pulp canal and contents being either a non-conductor or a poor conductor will contrast very sharply with the normal conductivity of the tissues surrounding apex of the root, so that under proper electrical arrangement we can detect the instant a broach, for instance, passes through the apical foramen, even much more quickly and accurately than can be told by the patient.

The appliances necessary for this are two or three dry cells, a milliammeter such as was used in cataphoresis, a negative electrode to be placed upon the gums opposite the root apex, and a fine insulated broach attached to the positive wire. The negative electrode is made hair-pin shape with rubber polishing cups mounted upon each end filled with cotton saturated with a sodium chloride solution.

The tension between the cups is sufficient to hold the appliance in position upon the gums. This electrode is placed nearest the root apex rather than the wrist because of the very small voltage required and therefore practically painless. If placed upon the wrist a very much higher voltage is necessary and any fluctuations will be felt. When cataphoresis was so largely used dentists frequently made the mistake of placing the negative electrode upon the wrist rather than upon the cheek to be held in place by the rubber dam.

When these are connected up the broach is introduced into the pulp canal and slowly worked to and perhaps through the apical foramen. If the ammeter is watched it will be observed that the index finger will make certain movements which when understood will be a reliable guide as to the depth of penetration of the broach with relation to the apical foramen. If the pulp canal is dry or filled with alcohol there will be no movement of the ammeter finger as the broach passes up the pulp canal because the alcohol is a non-conductor of electricity, but just as the broach reaches the foramen the finger will suddenly move over several points, due to the fact that an electrical circuit has been established from the broach through the moist tissues surrounding the root apex. Normal animal tissues and pus are conductors of electricity.

While the greatest contrast and widest movement of the index finger is observed in a perfectly dry or alcohol filled canal, yet if closely watched, a root canal filled with a fluid conductor such as water or the Dakin solution will show sufficient contrast to be reliable. The index finger will waver when the broach is first introduced into the canal, gradually moving over the scale as the broach approaches the end of the root, but just as it emerges from the foramen the index finger will suddenly move to the right several points. This is due to the fact that the periapical tissues and fluids are better electrical conductors than the pulp canal contents. Bear in mind, we have theoretically an hour glass to deal with where the apical foramen represents the hole in the hour glass. The metallic broach is a good conductor which forms half of the glass and the comparatively large amount of conductive tissue between the apex and the sponge electrode is the other half of the hour glass.

When the broach makes connection with the periapical tissues it is like puncturing the hole in the hour glass—the sand begins to run.

As previously stated, but two or three dry cells are necessary and if the tooth is pulpless there will be no pain, although if three cells are used the patient can indicate the moment the broach emerges from the foramen by reason of a very slight electrical shock, but not enough to be really painful.

The second method of getting the root length is one making use of the X-ray. This is more particularly for use at the time of filling the root and to be filed away for future reference. It was found that out of many hundred teeth measured that the following was the average length in millimeters of the single rooted teeth. An inch is approximately twenty-six millimeters long:

Superior.—Central, 21 millimeters; lateral, 22 millimeters; cuspid, 24 millimeters; bicuspid, 20 millimeters.

The corresponding teeth of the lower jaw averaged one millimeter less in length. We thus see than the various lengths of the single rooted teeth range between twenty and twenty-five millimeters. I therefore devised a set of six steel broaches made with short brass handles thereon placed exactly twenty to twenty-five millimeters from the point of the broach.

The handles were grooved with from one to five grooves thereon according to the length of the broach. The shortest with no groove at all indicating that that broach is just twenty millimeters long from the handle to the point and the broach with five grooves thereon indicates than that broach is just twenty-five millimeters long. The other lengths were arranged between.

The operator selects the broach as near the length of the root as he can judge and inserting it in the tooth till the shoulder of the handle comes against the end of the tooth, skiagraphs the tooth with the broach in situ. The grooves on the handle indicate just what length of broach was used. This will then tell just how long that tooth is as compared with the marked broach, which may or may not have reached the apex, but he now has by comparison an exact measure of the root for immediate use and for future reference.

While the schemes here presented may seem very elementary, we can not deny the fact that the dentist must check up his root canal work. We have guessed long enough. While they apply especially to single rooted teeth we shall from time to time find them applicable to multi-rooted teeth.

